# Determination of Bioactive Compound Kynurenic Acid in *Linum usitatissimum* L.

**DOI:** 10.3390/molecules29081702

**Published:** 2024-04-09

**Authors:** Magdalena Wróbel-Kwiatkowska, Waldemar Turski, Grażyna Silska, Magdalena Rakicka-Pustułka, Lucyna Dymińska, Waldemar Rymowicz

**Affiliations:** 1 Department of Biotechnology and Food Microbiology, Wrocław University of Environmental and Life Sciences, Chełmońskiego 37, 51-630 Wroclaw, Polandwaldemar.rymowicz@upwr.edu.pl (W.R.); 2 Department of Experimental and Clinical Pharmacology, Medical University of Lublin, Jaczewskiego 8B, 20-090 Lublin, Poland; waldemar.turski@umlub.pl; 3 Institute of Natural Fibres and Medicinal Plants—National Research Institute, Wojska Polskiego 71B, 60-630 Poznań, Poland; grazyna.silska@iwnirz.pl; 4 Department of Bioorganic Chemistry, Faculty of Production Engineering, Wroclaw University of Economics and Business, Komandorska 118/120, 53-345 Wroclaw, Poland; lucyna.dyminska@ue.wroc.pl

**Keywords:** kynurenic acid (KYNA), flax, HPLC

## Abstract

Kynurenic acid (KYNA) is a bioactive compound exhibiting multiple actions and positive effects on human health due to its antioxidant, anti-inflammatory and neuroprotective properties. KYNA has been found to have a beneficial effect on wound healing and the prevention of scarring. Despite notable progress in the research focused on KYNA observed during the last 10 years, KYNA’s presence in flax (*Linum usitatissimum* L.) has not been proven to date. In the present study, parts of flax plants were analysed for KYNA synthesis. Moreover, eight different cultivars of flax seeds were tested for the presence of KYNA, resulting in a maximum of 0.432 µg/g FW in the seeds of the cultivar Jan. The level of KYNA was also tested in the stems and roots of two selected flax cultivars: an oily cultivar (Linola) and a fibrous cultivar (Nike). The exposure of plants to the KYNA precursors tryptophan and kynurenine resulted in higher levels of KYNA accumulation in flax shoots and roots. Thus, the obtained results indicate that KYNA might be synthesized in flax. The highest amount of KYNA (295.9 µg/g dry weight [DW]) was detected in flax roots derived from plants grown in tissue cultures supplemented with tryptophan. A spectroscopic analysis of KYNA was performed using the FTIR/ATR method. It was found that, in tested samples, the characteristic KYNA vibration bands overlap with the bands corresponding to the vibrations of biopolymers (especially pectin and cellulose) present in flax plants and fibres.

## 1. Introduction

**Figure 1:**
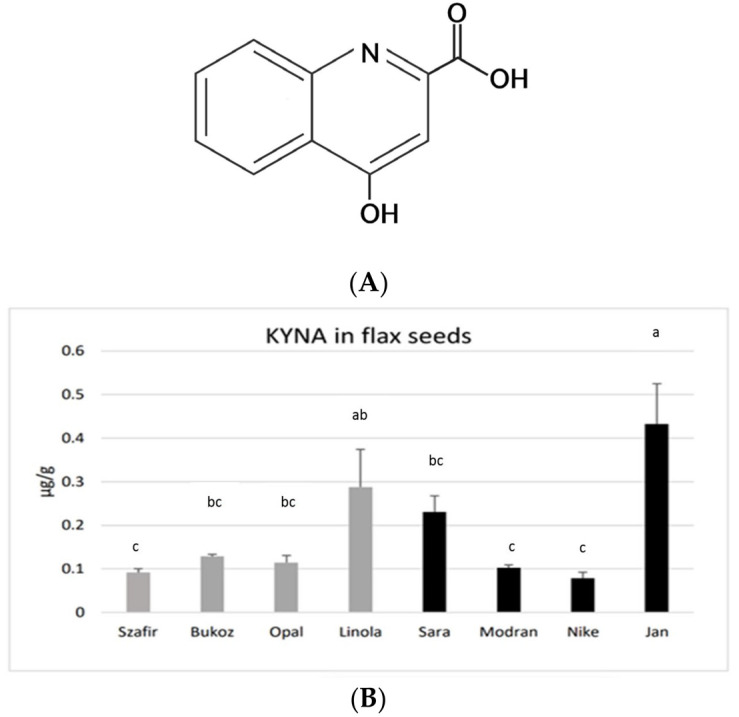
(**A**). The chemical structure of KYNA. (**B**). KYNA content in flax seeds derived from eight flax cultivars grown in Poland. The oily cultivars (Szafir, Bukoz, Opal, Linola) and fibre cultivars (Sara, Modran, Nike, Jan) were analysed. KYNA was determined by the HPLC method, as described in the Materials and Methods Section (Section 3) and expressed in μg/g FW. Error bars represent standard deviation. Letters (a–c) designations represent significant differences between cultivars.

Kynurenic acid (C_10_H_7_NO_3_, 4-hydroxyquinoline-2-carboxylic acid, KYNA) (Figure 1A) is an interesting bioactive compound with multiple health-beneficial activities in mammals [1].

KYNA has been proven to have positive effects on the digestive system [1] and the nervous system [2]. KYNA has been found to be an effective neuroprotective [3] and antimicrobial agent [4]. Inappropriate enzyme activity in the KYNA pathway is the source of neurological diseases [5]. KYNA is as an agonist of the GPR35 receptor may have an impact on the immune system, cardiovascular system, adipocytes and inflammation [2,6,7]. It was also reported that the level of KYNA may be a crucial factor in diseases (e.g., obesity and dysfunction of the vascular system) [8,9]. Since obesity and cardiovascular diseases constitute increasing health problems in developing societies, it is justifiable to search for natural products that are effective in the prevention and treatment of these diseases. On the other hand, it was stated that KYNA improves the healing of skin wounds [10,11,12] and prevents the formation of scars [10]. Thus, two aspects of KYNA application should be considered: its systemic effect on the human body (e.g., on the digestive system) and its local action (e.g., for wound healing).

In animals, KYNA is produced from tryptophan along the kynurenine pathway, but there is still no evidence for the presence of an analogous pathway in plants. KYNA is a hydroxy acid whose presence has been proven in many animal tissues and organs, including the liver, kidney and brain [13,14]. KYNA synthesis has also been demonstrated in yeast: *Saccharomyces cerevisiae*, *S. pastorianus* [15] and the unconventional yeast *Yarrowia lipolytica* [16,17]. It was also reported that the presence of KYNA was confirmedin plants (e.g., tobacco, nettle, potato, dandelion and plants of the genus *Ephedra*), but the concentrations of KYNA measured in plant tissues were low and depended on the plant species and development stage [18,19]. It is suggested that KYNA in plants is synthesized in a kynurenine pathway, similar to that in mammalian cells [4]. This suggestion may be confirmed by the fact that, in the genome of some plants, including rice (*Oryza sativa* L.), sequences of genes encoding enzymes from the kynurenine pathway were identified, i.e., genes encoding tryptophan dioxygenase, arylformamidase, kynurenine 3-monooxygenase, kynureinase and 3-hydroxyanthranylate 3,4-dioxygenase [20]. It was stated that the obtained sequences showed 24–41% similarity to yeast and animal sequences. It should be also pointed out that the precursor of KYNA, namely tryptophan, is natively synthesized in plants, but not in mammals. The significance of the kynurenine pathway for plants may be connected to nicotinamide adenine dinucleotide (NAD) biosynthesis, which can be produced from its precursor, aspartic acid, or alternatively from tryptophan [20].

Currently, little is known about the importance of KYNA in plant physiology; however, it has been mentioned that kynurenine (KYN) and KYNA can inhibit auxin biosynthesis [21]. KYN has been proven to negatively affect embryogenesis in *Hordeum vulgare* L. tissue cultures [22]. The level of KYNA found in plants eaten as vegetables, e.g., cauliflower, broccoli and potatoes, was low, in the range 0.04–0.41 µg/g FW [1]. In the case of potato cultivars with yellow- (Ismena) and purple-flesh tubers (Provita), the KYNA level was slightly higher, at 0.226 and 0.683 µg/g FW (fresh weight) [23]. Interestingly, the thermal processing of potato tubers caused a reduction in the KYNA concentration.

Recent research showed that KYNA may also be synthesized in the industrially important plant hemp (*Cannabis sativa* L.), and the KYNA content in hemp leaves was 18.6 µg/g dry weight (DW) [24].

Finally, it can be assumed that our diet is rather poor in KYNA. In fact, the richest source of this compound is the unusual food product chestnut honey (containing 2.1 mg/g KYNA) [25]. The presence of KYNA in flax has not been determined so far. Therefore, it is interesting to evaluate whether KYNA is present, in flax seeds, which are consumed by humans for their health-promoting effects, but also in flax stems—the source of fibre used by the textile, construction and pharmaceutical industries.

Flax (*Linum usitatissimum* L.) is an annual plant that has been cultivated since ancient times. It is a multipurpose crop, delivering valuable fibres for the production of textiles and composites and oil for the cosmetic and food industries [26]. Flax oil and seeds are among the richest sources of essential unsaturated omega-3 α-linolenic fatty acid [27], and moreover they contain lignans, sterols, essential amino acids, minerals and vitamins [28]. Thus, flax seeds and oil are considered functional foods because they are a source of nutrients and have a positive effect on human health. However, the second product from this plant—flax fibre—can also be used as a component of biocomposites with a polypropylene, polylactide or polycaprolactone matrix for potential application in medicine and agriculture [29]. These fibres can also be used as a component of a new dressing for medical purposes [30], especially after plant genetic engineering and enrichment with bioactive compounds (e.g., PHB, poly-β-hydroxybutyrate).

Thus, the aim of the present study was to investigate the presence and content of KYNA in flax. An additional research goal was to determine whether KYNA synthesis from its precursors, tryptophan and kynurenine, occurs in the plant. To avoid the impact of any external factor, experiments were conducted on flax seedlings grown under aseptic in vitro conditions.

## 2. Results and Discussion

### 2.1. KYNA Level in Flax Seeds

KYNA was found in the seeds obtained from all investigated flax cultivars, grown earlier under field conditions. The highest amounts of KYNA were detected in the fibrous cultivar Jan (0.43 µg/g FW) and in the oily cultivar Linola (0.29 µg/g FW). A moderate content of KYNA was found in the fibrous cultivar Sara (0.23 µg/g FW). A lower KYNA level of about 0.1 µg/g FW was detected in the other tested cultivars (Figure 1B). the assessed concentrations of KYNA are distinctly lower than those noted for other known plant-derived food constituents consumed by humans, e.g., leaves of basil (14.08 µg/g) and thyme (8.87 µg/g), as well as other spices and herbs [31]. Flax seeds showed a similar level of KYNA as was measured for the fruits of black pepper (0.1 µg/g) or for raw potato tubers (0.226–0.683 µg/g FW) [24]. However, potato tubers may only be consumed after thermal processing, and this process significantly decreases the level of KYNA. On the other hand, spices (black pepper, basil, thyme, etc.) are consumed in very low amounts; hence, the observed concentrations of KYNA in flax seeds (up to 0.43 µg/g FW) seem to be a good result, especially as they can be consumed in the raw form, as flour or as whole seeds. The obtained data raise a question about the KYNA level in flax oil, which will be verified in the future.

### 2.2. KYNA Levels in Flax Plants Grown in Tissue Cultures

All the measurements were performed in plant material derived from aseptic flax tissue cultures of two chosen flax cultivars: fibrous Nike and oily Linola. These cultivars were chosen because they were initially used for flax transformation: cultivar Nike for the generation of M transformants, enriched via the genetic engineering method in poly-β-hydroxybutyrate (PHB) [32], and the Linola cultivar for the generation of W transformants with increased flavonoid content [33]. Then, the fibres and seeds derived from field-cultivated transformed flax plants were used as the components of flax bandages applied for wounds [34].

**Figure 2:**
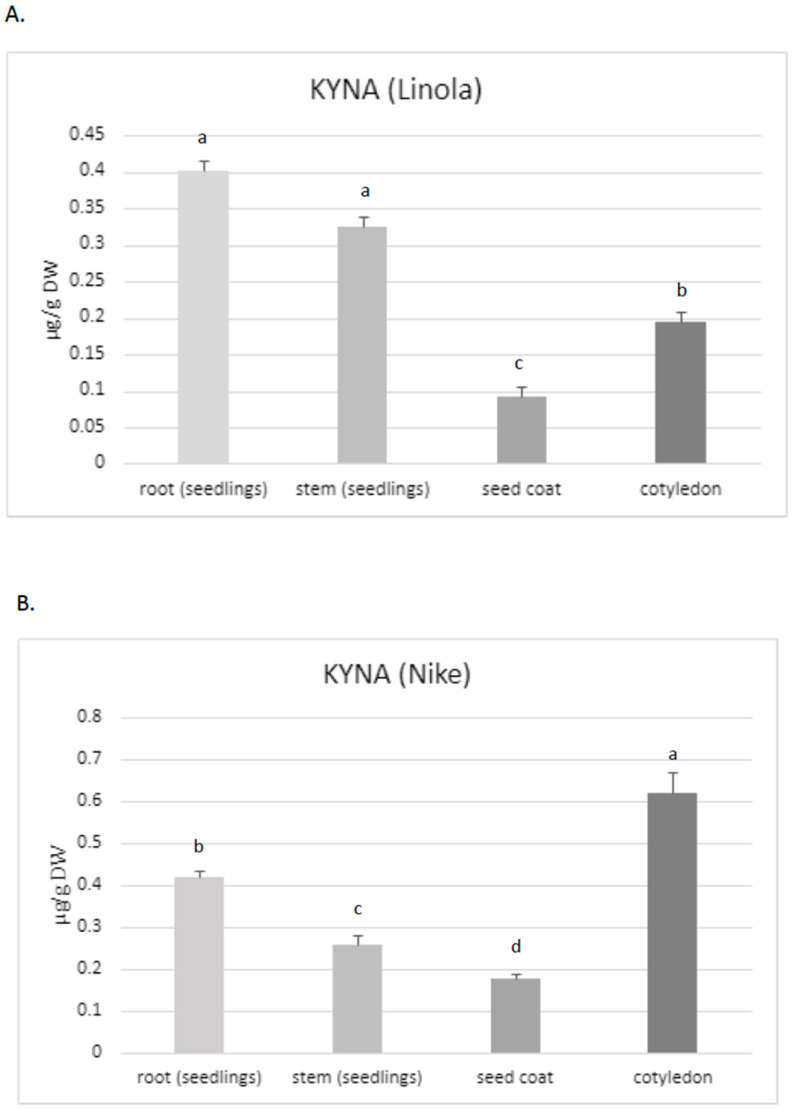

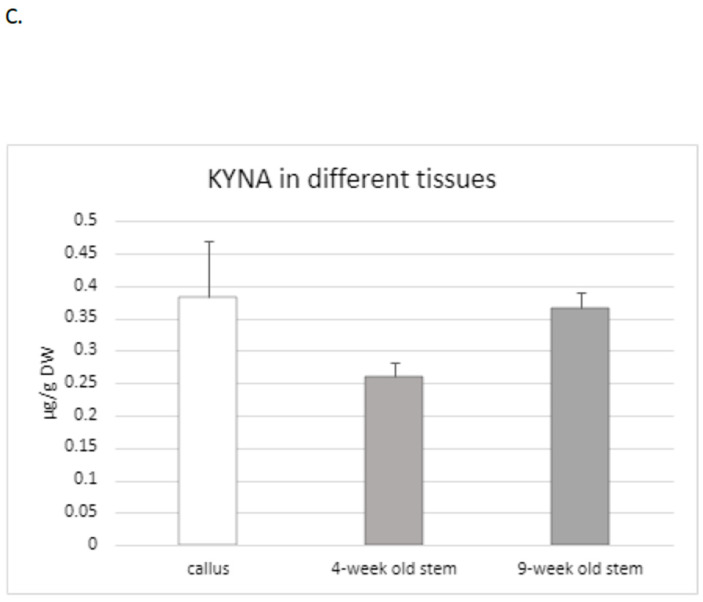
KYNA content in different organs of flax cultivars Linola and Nike. (**A**). The level of KYNA measured in different organs of flax plants cv. Linola (KYNA was determined in 10–14-day sterile seedlings, derived from in vitro cultures); (**B**). KYNA content determined in individual organs of plants belonging to the Nike cultivar (KYNA was isolated and analysed in 10–14-day sterile seedlings grown in tissue cultures); (**C**). Determination of KYNA content in callus initiated on explants obtained from Nike cultivar. The effect of plant age on KYNA content (the metabolite was measured in 4-week-old and 9-week-old flax stems cv. Nike, derived from sterile tissue cultures). KYNA was determined by the HPLC method, as described in the Materials and Methods Section. Error bars represent standard deviation. Letters (a–d) designations represent significant differences between flax organs.

Thus, the aim of the present study was to assess the KYNA content, especially in these two flax cultivars, which are characterized by their high morphogenetic abilities in tissue culture and potential application in medicine. The obtained data confirmed that KYNA is present in each part of the flax plants. The roots of Linola and Nike contained higher KYNA levels (0.40 µg/g and 0.42 µg/g DW, respectively) than stems (0.325 µg/g and 0.26 µg/g DW, respectively) and the seed coat (0.09 µg/g and 0.17 µg/g DW, respectively) (Figure 2A,B). However, KYNA content in the roots was only higher than that in the stems for one tested cultivar (Nike) (these changes were statistically important); for cultivar Linola, the elevated level was not statistically important. The content of KYNA in cotyledons was also dependent on the cultivar, i.e., in Linola and Nike cotyledons, it was 0.195 µg/g DW and 0.62 µg/g DW, respectively.

Notably, the content of KYNA in flax stems was at a similar level as detected in the leaves for other plants, e.g., greater celandine *Chelidonium majus* L. (0.283 µg/g FW), and lower than that detected in horse-chestnut leaves (0.636 µg/g FW) [35]. However, the level of KYNA noted for flax roots was higher than that measured for other plants, e.g., in dandelion (0.05 µg/g DW) [35]. For one tested cultivar (Nike), it was noticed that the flax roots contained statistically higher amounts of KYNA than stems (shoots and leaves). Diverse observations were made for hemp, for which the highest KYNA level was determined in the photosynthetic organs (i.e., leaves) [24]. The maximum amounts of KYNA determined in hemp leaves were higher than those found in flax. The content of KYNA in the hemp leaves of plants grown in soil and cultured in hydroponic systems was 18.6 µg/g and 8.1 µg/g DW, respectively. According to Russo et al. (2022), the KYNA amount was dependent on the cultivation system and age of plants [24]. It should be noted that the present study investigated plants from tissue cultures; a quantitative determination of KYNA in soil cultured plants would be very interesting and could answer the question about the application significance of flax plants and fibres in the context of the KYNA source. It should be noted that the KYNA concentration in hemp roots obtained from soil culture was under the limit of quantification, while in the hydroponic system it was 0.8 µg/g DW. Thus, hemp contained two-fold higher levels of KYNA in the roots when compared to flax roots. Interestingly, the level of KYNA in flax stems was similar to that quantified in hemp stems (0.5 µg/g DW) [24]. However, the cultivation systems of these two plants (hemp, flax) were different, and this may be the reason for the observed differences.

Because the level of KYNA in the stems and roots of two tested flax cultivars (Nike, Linola) was comparable, for the next experiments only the Nike cultivar was chosen. It was found that the KYNA level measured in flax stems was dependent on the age of the plant, i.e., older stems (9 weeks old) of the Nike cultivar contained 40% more KYNA than the younger stems (4 weeks old) (Figure 2C).

KYNA was also analysed in callus tissue, which is critical to establish a suspension culture and remains the basis for many biotechnological applications of plants [36]. The induced callus, derived from explants of the Nike cultivar, was green and compact and did not possess friable properties. The level of KYNA determined in the callus was equal to 0.38 ± 0.08 µg/g DW and was higher than that measured in 9-week-old stems of the same cultivar (Figure 2C).

This observation may suggest that the callus could be considered as an efficient tissue for KYNA synthesis, but further analyses, especially with callus suspension cultures, should be performed in the future.

### 2.3. KYNA Synthesis in Flax Exposed to Tryptophan and Kynurenine

It is widely considered that, in animals, KYNA is synthesized from kynurenine, which is formed from tryptophan in the kynurenine pathway by the following enzymes: tryptophan 2,3-dioxygenase or indoleamine 2,3-dioxygenase and kynurenine formamidase. In the next step, kynurenine is metabolized by kynurenine amino transferases (KATs) to KYNA [37]. To investigate whether flax utilizes tryptophan or kynurenine to synthesize KYNA, both precursors were added to the standard medium used for flax cultivation and KYNA content was determined in plants’ roots and stems.

The effect of tryptophan and kynurenine on flax development and biomass growth was also estimated, since tryptophan is a precursor for auxin biosynthesis in plants (i.e., tryptophan-dependent auxin synthesis). Auxins are known plant growth regulators with an essential role in the processes of plant growth and development [38,39,40,41], while kynurenine was reported to inhibit auxin biosynthesis in plants [42].

**Figure 3:**
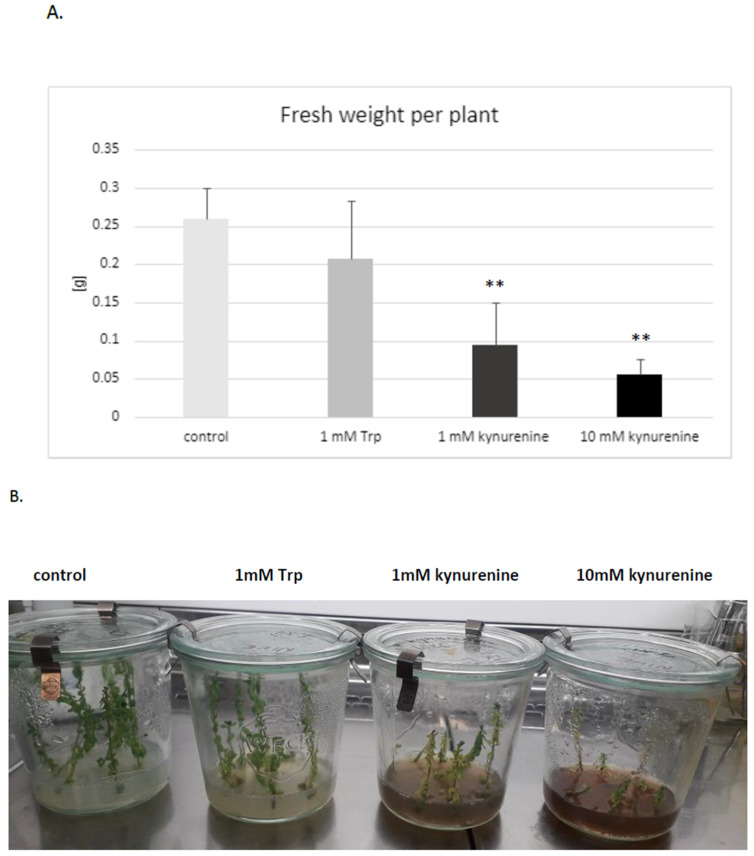
Effect of KYNA precursors’ (tryptophan and kynurenine) addition in the medium on the biomass of flax grown in tissue cultures. (**A**). Fresh weight of flax biomass (in grams) is presented on the vertical axis. Trp—tryptophan; error bars represent standard deviation. Asterisks indicate a statistically significant effect, tested with Student’s *t*-test; ** *p* < 0.01. (**B**). The phenotype of flax plants (cultivar Nike) exposed to the KYNA precursors.

It was established that both doses of kynurenine (1 and 10 mM) in the medium negatively affected flax development; plants exposed to a high concentration of kynurenine did not develop roots. The biomass was also distinctly decreased in comparison to control plants cultured under the same conditions without any supplement (Figure 3A). Therefore, for further analyses, only a lower concentration of kynurenine (1 mM) was applied.

Similarly, the presence of tryptophan in medium in both concentrations (1 mM, 10 mM) reduced or blocked root formation. It should be stated that some of the flax plants cultured in the medium with tryptophan had no roots. The reason for this phenomenon might be the redirection of tryptophan to auxin biosynthesis, because it was shown that an increased auxin level also inhibits root growth [43,44]. Thus, higher levels of auxin, which are the main regulators of rooting, may be the direct reason for the observed inhibition of root formation in plants treated with auxin precursors (tryptophan). It should also be pointed out that the exogenous treatment of plants with auxins may also cause an inhibition of root growth [44].

It was reported that kynurenine is an alternative substrate for L-tryptophan aminotransferase, a crucial enzyme for auxin synthesis, which catalyses the conversion of tryptophan to indolyl-3-pyruvic acid [42]. Thus, high concentrations of kynurenine can inhibit auxin synthesis. The mechanism of kynurenine’s impact on plant growth may also result from its effect on responses to auxins.

**Figure 4:**
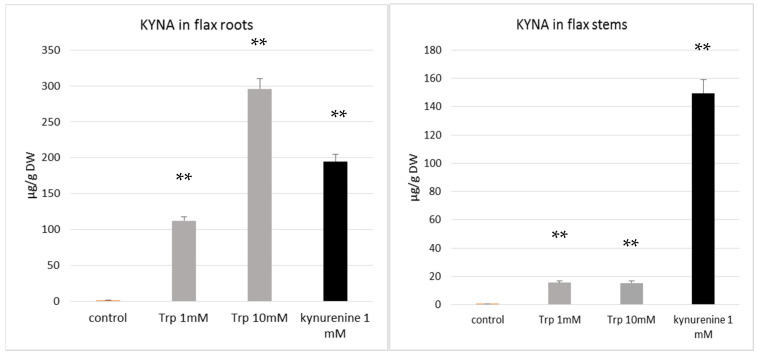
Effect of KYNA precursors (tryptophan and kynurenine) on kynurenic acid content, measured in roots and stems of flax (cv. Nike) cultivated in vitro. (**A**). KYNA content in roots of flax cultivated in the presence of tryptophan (1 mM, 10 mM) and kynurenine (1 mM); (**B**). KYNA content in stems of flax cultivated in the presence of tryptophan (1 mM, 10 mM) and kynurenine (1 mM). Trp—tryptophan. KYNA was determined by the HPLC method, as described in the Materials and Methods Section. Error bars represent standard deviation. Asterisks indicate a statistically significant effect tested with Student’s *t*-test; ** *p* < 0.01.

The results of our study indicate that flax growing in the presence of tryptophan and kynurenine contains more KYNA than control plants (Figure 4A,B). This finding supports the hypothesis that both of the added precursors, tryptophan and kynurenine, were taken up by the plant and used to synthesize KYNA. The data confirm previous results showing that the KYNA precursor kynurenine may be absorbed by plants [35]. It was also found that the flax plant’s response to the tested precursors was different for various organs, such as the stems and roots. Kynurenine was a more efficient substrate for KYNA synthesis in plant stems, resulting in a KYNA level of 149.5 µg/g DW, while the supplementation of medium with tryptophan more effectively increased KYNA synthesis in roots (Figure 4A,B). It can be assumed that the KYNA level increased from 1.42 µg/g DW in control roots (not exposed to tryptophan) to 295.9 µg/g DW in roots growing in the medium supplemented with 10 mM tryptophan. The obtained results indicate that the synthesis of KYNA from tryptophan and kynurenine occurs in flax. The present data are consistent with previous results indicating that KYNA in plants may be synthesized from kynurenine [35]. Turski et al. analysed another plant (dandelion) and also stated that, in the presence of kynurenine, the highest level of KYNA content was accumulated by green tissue (leaves) [35]. Unfortunately, they did not investigate the influence of tryptophan on KYNA production in the plants. The role of KYNA in plant physiology is still unknown, and will be studied in the future.

Since KYNA has established health-promoting properties but its content in food is relatively low [1], a search to identify rich sources of natural KYNA is in progress. The obtained results indicate that the tested flax cultivars contained a maximum of 0.4 µg/g FW KYNA in the seeds. Flax tissue cultures can be considered as an efficient platform for KYNA production. In this context, the efficient production of KYNA, especially from tryptophan, seems very promising. Further experiments will be carried out in the future to assess the potential of callus suspension cultures initiated from flax for KYNA synthesis. It should be pointed out that KYNA is a beneficial compound in wound healing, since it was reported that KYNA prevents scarring and may be used as an anti-fibrogenic agent [10]. It was proved that kynurenine and its derivatives (e.g., KYNA) might be considered as effective wound healing agents in a rabbit ear scar model [11,12] and in clinical human trials [10]. In this regard, the presence of KYNA in flax plants is an interesting issue, because this plant is a source of industrially important fibre that can be used in medical devices (bandages) for wound healing [26,34].

### 2.4. Spectroscopic Characterization of KYNA and Investigation of KYNA Using FTIR Method

The FTIR spectra of flax plants and flax fibres of the Nike cultivar confirm the presence of biopolymers, the components of plant cell walls. The general shape of these spectra is typical for the cellulose spectrum, enriched with lignin and pectin bands.

**Figure 5:**
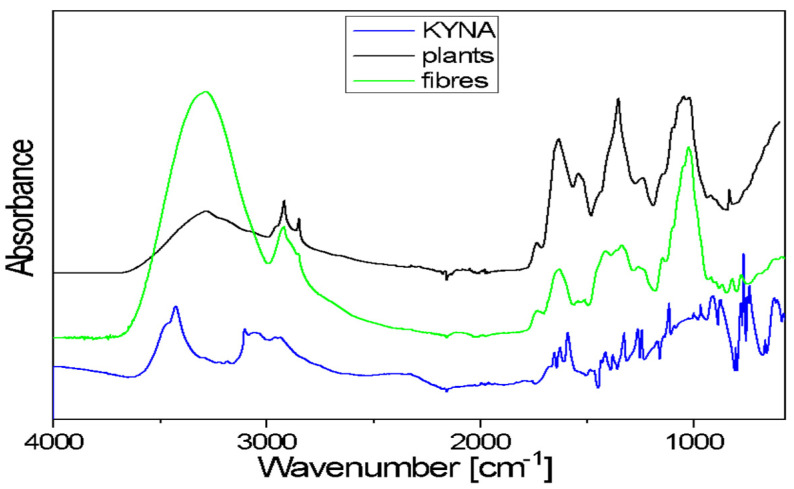
FTIR spectra of the studied samples: kynurenic acid (KYNA), flax plants and fibres of cultivar Nike.

Kynurenic acid is a chemical compound belonging to hydroxy acids. It is composed of fused benzene and pyridine rings with a hydroxyl and carboxyl group on the pyridine ring. The 3700–3200 cm^−1^ range in the FTIR spectrum of KYNA is characteristic of the ν(OH)-stretching vibrations of free hydroxyl groups and those involved in hydrogen bonds. The broad band, with a 3200–2600 cm^−1^ range, corresponds to the asymmetrical and symmetrical stretching vibrations of =C–H bonds of aromatic rings. A broad band in the 3700–3000 cm^−1^ range is also observed in the plants and fibres’ spectra. This band corresponds to the stretching mode of the hydroxyl groups taking part in the formation of intra- and inter-molecular hydrogen bonds in cellulose. These spectra exhibited two bands at around 2900 and 2850 cm^−1^, characteristic of the asymmetrical and symmetrical stretching vibrations of methylene groups of cellulose. In the 1730–1500 cm^−1^ range, the observed bands occur at 1670, 1650, 1626 and 1590 cm^−1^. These multiples correspond to the stretching vibrations of the carboxyl groups and the stretching vibrations of benzene and pyridine rings present in KYNA. In both analysed samples (flax plants and fibres), the contour in the 1780–1550 cm^−1^ range is the characteristic area for pectin vibrations (Figure 5).

The contour in the 1480–1180 cm^−1^ region of flax plants and fibres spectra consists of several bands that correspond to the following bending vibrations: δ(CH), mixed δ(OH⋅⋅⋅O) + δ(CH2) vibrations or ω(CH2) vibrations of biopolymers. The KYNA spectrum in the same range contains bands at the following wavenumbers: 1414, 1378, 1321, 1260 and 1244 cm^−1^. These bands are characteristic of the stretching vibrations of the pyridine and benzene rings and in-plane bending vibrations of CH and OH groups. The main bands observed in the 1180–840 cm^−1^ region of flax plants and fibres spectra correspond to the following vibrations: stretching of the glycosidic ring mode; stretching symmetric and asymmetric C–O–C mode of the β(1→4) glycosidic linkage coupled with the ring-stretching or ring-bending mode. In the same range of the KYNA spectrum, there are bands at wavenumbers 1114, 1004, 967, 916 and 870 cm^−1^. These bands correspond to the bending and stretching vibrations of aromatic rings, stretching vibrations ν(C–N) of the pyridine ring, stretching vibrations C-OH and in-plane bending vibrations of CH groups.

A broad band of 600 cm^−1^ is observed in the spectra of both studied samples (flax plants and fibres). This corresponds to the out-of-plane bending γ(OH⋅⋅⋅O) modes of the hydroxyls engaged in the O–H⋅⋅⋅O hydrogen bonds in cellulose. The KYNA spectrum in the 830–600 cm^−1^ range contains bands at wavenumbers 802, 776, 765, 745, 738 and 663 cm^−1^. These bands are characteristic of the in-plane and out-of-plane bending vibrations of the pyridine and benzene rings and in-plane and out-of-plane bending vibrations of the hydrogen bonds.

In sum, the bands that are characteristic of KYNA vibrations overlap with the bands corresponding to the vibrations in biopolymers present in flax plants and fibres. A spectroscopic analysis of the extracts was planned to determine the KYNA content in biological material.

## 3. Materials and Methods

### 3.1. Plant Material

The Polish cultivars of flax (*Linum usitatissimum* L.) Opal, Szafir, Bukoz, Modran, Nike, Sara, Jan and the Canadian cultivar Linola were used in the present study. The used cultivars included oily cultivars (Opal, Szafir, Bukoz, Linola) and fibre cultivars (Modran, Nike, Sara, Jan). All Polish cultivars were derived from field cultivation carried out in 2018 by the Institute of Natural Fibres and Medicinal Plants, Poznań, Poland. All are described in the National List of Agricultural Plant Varieties, 2019. It was confirmed that all the methods were performed in accordance with the relevant legislation. Flax fibres (Nike cultivar) were obtained from the Department of Genetic Biochemistry, Faculty of Biotechnology, University of Wroclaw.

### 3.2. Plant Tissue Cultures

The plants were cultured in a phytotron under a regime of 16 h light at 21 °C in the day and 8 h dark at 16 °C in the night, with 65% relative humidity. The flax plants were grown on MS medium [45], supplemented with 1% sucrose and 0.8% agar. The pH of the medium was 5.8. Media were autoclaved at 121 °C for 20 min. Plant Preservative Mixture (PPM, Plant Cell Technology, Washington, DC, USA), at a concentration of 750 μL/1 L, was added to the medium to prevent microbial contamination and ensure sterile conditions during plant growth.

### 3.3. Flax Seedlings

The seed germination of the two selected cultivars of flax, Nike and Linola, was performed in a phytotron in the darkness. After 10–14 days, young seedlings were obtained and exposed to a 16 h light (21 °C)/8 h dark (16 °C) regime. Then, flax stems, roots, seed coat and cotyledons were harvested, dried (50 °C, 24 h), pulverized in a mortar and analysed for the presence of KYNA.

### 3.4. Callus Induction on Flax Explants

Young seedlings of the cultivar Nike, derived as the result of seed germination, were used for callus formation. To achieve this aim, explants (hypocotyls and cotyledons) were transferred to callus induction medium (MS medium with 2.5% glucose, 2.5% sucrose, 1 mg/L BAP, 0.05 mg/L NAA, 0.8% agar) [46]. The explants were cultured and transferred to the fresh medium every 7 days. The induced calli were harvested, dried at 50 °C for 24 h and analysed for KYNA level.

### 3.5. Plant Material for Determination of Relationship between KYNA Content and Plant Age

The clonal propagated flax plants (cv. Nike) were grown for 4 weeks and, alternatively, 9 weeks in the MS medium, with the addition of 1% sucrose and 0.8% agar in the sterile tissue cultures (method was described above). Then, the tissue was harvested, dried at 50 °C and analysed for KYNA level via the HPLC technique.

### 3.6. Supplementation of Medium with KYNA Precursors

The explants (stem segments, without roots) of 4-week-old flax plants (cultivar Nike) were transferred to the MS medium (with 1% sucrose, 0.8% agar) supplemented with L-tryptophan (1 mM or 10 mM) or, alternatively, with L-kynurenine (1 mM or 10 mM). Both chemicals were purchased from Sigma-Aldrich and the applied concentrations were chosen based on the published data [35]. Flax plants (cv. Nike), grown under the same conditions without tryptophan and kynurenine, served as the control. After 1 month of growth on medium containing KYNA precursors, the obtained plants (stems and roots) were harvested, dried at 50 °C for 24 h and analysed for the presence of KYNA [47].

### 3.7. KYNA Extraction and Measurement via High-Performance Liquid Chromatography (HPLC) Method

The quantitative determination of KYNA was performed as described by Turski et al. [31,35]. The samples of dried, pulverized plant material or flax seeds were mixed with distilled water (1:10, *w*/*v*) and homogenized. In the case of KYNA isolation from flax seeds, they were ground in a laboratory mill (ZBPP P008M Sp. z o.o. Bydgoszcz, Poland) before the procedure. After centrifugation (5000 rpm, 10 min), the obtained supernatant was acidified with trichloroacetic acid (50%) and centrifuged (12,000 rpm, 10 min). Then samples were mixed with 0.1 N HCl and the fraction containing KYNA was separated using ion exchange chromatography with Dowex 50 columns and prewashed with 0.1 N HCl. Then, the column was washed using 1 mL of 0.1 N HCl and 1 mL of distilled water, and KYNA elution was performed with distilled water (3 mL). KYNA level was analysed using the HPLC method (Dionex HPLC system; C18 reverse-phase column) and quantified fluorometrically (Dionex RF2000 fluorescence detector; excitation 350 nm; emission 404 nm). The mobile phase consisted of 50 mM sodium acetate and 250 mM zinc acetate (pH 6.2) containing 5% acetonitrile. The flow rate was 1.0 mL/min. KYNA purchased from Sigma was used as a reference for HPLC.

### 3.8. Infrared Measurements

FTIR/ATR spectra for flax plants of cultivar Nike, Nike fibres and the standard of KYNA were recorded in the 4000–550 cm^−1^ range using a Nicolet 6700 spectrometer (Thermo Fisher Scientific, Waltham, MA, USA) with a portable ATR set. The resolution of these measurements was 2 cm^−1^.

### 3.9. Determination of Fresh Weight of Plants

Flax plants were collected from sterile in vitro cultures. The fresh weight was measured using an electric balance and expressed as the mean value for 9–11 plants.

For KYNA measurements, flax plants were grown under the same conditions as mentioned above, then collected and air-dried at 50 °C for 24 h.

### 3.10. Statistical Analysis

The results were obtained as the averages of independent replicates ± standard deviations. For the results presented in Figure 1B and Figure 2, a one-way analysis of variance (ANOVA, St. New Providence, NJ, USA) was applied, combined with Duncan’s test [48]. Statistica 13 (TIBCO Software, Inc., Palo Alto, CA, USA) software was used. For the results presented in Figure 3 and Figure 4, a statistical analysis was prepared using Student’s *t*-test (* *p* < 0.05; ** *p* < 0.01).

## 4. Conclusions

In this study, we determined the presence of KYNA, an interesting bioactive compound that exhibits health-promoting properties, in flax seeds and flax tissue cultures.

The measured KYNA level was dependent on the analysed flax cultivar and organ and tissue age. The KYNA amounts observed in the seeds were in the range 0.07–0.43 µg/g, and the highest level was observed for the Jan cultivar (0.43 µg/g). For the cultivar Linola cultivar, the highest level of KYNA was noted in the roots, while for the Nike cultivar, the highest amount of KYNA was measured in cotyledons. Finally, both cultivars showed a similar amount of KYNA in roots and stems. It was also found that older plants accumulated about 40% more KYNA. In addition, data demonstrating that KYNA can be synthesized from tryptophan and kynurenine were presented. The flax organs exhibiting the highest KYNA level (295.9 µg/g DW) were roots derived from flax plants grown in tissue cultures in a medium supplemented with KYNA precursor (tryptophan). Furthermore, the possibility of cross-talk between the applied KYNA precursors (tryptophan, kynurenine) and auxin synthesis was discussed. The observed results that callus tissue exhibited slightly higher amounts of KYNA than 9-week-old stems, suggesting that callus suspension cultures might be a good system for KYNA synthesis, especially since such cultures can be used to enhance the production of the desired compounds. Since KYNA improves wound healing, the proposed flax suspension cultures may serve as a source of this important compound or/and as an ingredient in creams used for wounds and scars.

Finally, the obtained data indicate that flax seeds may be a good source of KYNA, especially as they can be consumed in their raw form, and that flax tissue cultures may be a sufficient method for KYNA synthesis in higher concentrations. However, this finding will be verified in the near future. KYNA was characterized by the FTIR/ATR method. A spectroscopic analysis of flax stems and fibres revealed that bands characteristic of pectin and cellulose overlap with KYNA vibrations. Thus, further analyses using extracts of stems and fibres will be performed in the future. It will be very interesting to quantitatively determine KYNA in whole plants and fibres, which remain in use as a product for medical devices (e.g., bandages).

## Data Availability

Data are contained within the article.
